# Distinct reinforcement learning profiles distinguish between language and attentional neurodevelopmental disorders

**DOI:** 10.1186/s12993-023-00207-w

**Published:** 2023-03-21

**Authors:** Noyli Nissan, Uri Hertz, Nitzan Shahar, Yafit Gabay

**Affiliations:** 1 Department of Special Education, University of Haifa, Haifa, Israel; 2 Edmond J. Safra Brain Research Center for the Study of Learning Disabilities, University of Haifa, 199 Abba Khoushy Ave, Haifa, Israel; 3 Department of Cognitive Sciences, University of Haifa, Haifa, Israel; 4 The School of Psychological Sciences, Tel Aviv University, Tel Aviv, Israel; 5 Sagol School of Neuroscience, Tel Aviv University, Tel Aviv, Israel

**Keywords:** Attention-deficit/hyperactivity disorder, Developmental dyslexia, Two-step task, Model-based vs. Model-free reinforcement learning

## Abstract

**Background:**

Theoretical models posit abnormalities in cortico-striatal pathways in two of the most common neurodevelopmental disorders (Developmental dyslexia, DD, and Attention deficit hyperactive disorder, ADHD), but it is still unclear what distinct cortico-striatal dysfunction might distinguish language disorders from others that exhibit very different symptomatology. Although impairments in tasks that depend on the cortico-striatal network, including reinforcement learning (RL), have been implicated in both disorders, there has been little attempt to dissociate between different types of RL or to compare learning processes in these two types of disorders. The present study builds upon prior research indicating the existence of two learning manifestations of RL and evaluates whether these processes can be differentiated in language and attention deficit disorders. We used a two-step RL task shown to dissociate model-based from model-free learning in human learners.

**Results:**

Our results show that, relative to neurotypicals, DD individuals showed an impairment in model-free but not in model-based learning, whereas in ADHD the ability to use both model-free and model-based learning strategies was significantly compromised.

**Conclusions:**

Thus, learning impairments in DD may be linked to a selective deficit in the ability to form action-outcome associations based on previous history, whereas in ADHD some learning deficits may be related to an incapacity to pursue rewards based on the tasks' structure. Our results indicate how different patterns of learning deficits may underlie different disorders, and how computation-minded experimental approaches can differentiate between them.

## Background

Developmental dyslexia (DD) and Attention-deficit/hyperactivity disorder (ADHD) are two of the most common neurodevelopmental disorders. Dyslexia is characterized by difficulties in acquiring reading, writing, and spelling skills, whereas ADHD is characterized by inattention, impulsivity, and hyperactivity symptoms. Traditionally, DD has been suggested to arise from phonological impairments [87] but domain-general accounts postulate sensory [46] or procedural learning impairments [65, 98, 99] in its etiology, thus providing a mechanistic account for the diverse range of linguistic and nonlinguistic symptoms observed in this disorder. ADHD has been associated with an executive function deficit [4], but a growing body of evidence points to key deficits in motivational/reward-related processes as well [7, 36, 37, 59, 69, 73, 77, 80, 89]. There is a high comorbidity between these two childhood neurodevelopmental disorders [105], including shared symptoms such as temporal processing impairments [22, 94], executive function deficits [56], and procedural learning deficiencies [1, 110, 34, 54, 57].

Despite decades of research, the neurocognitive basis of these two disorders is still highly debated and the reason for the overlap is not yet fully understood. Recent advances in the research of comorbidity prompt a change from single deficit models to multiple models of developmental neuropsychology. According to the multiple deficit model [70], there are multiple probabilistic predictors of neurodevelopmental disorders across levels of analyses and comorbidity arises due to shared risk factors.

Interestingly theoretical and empirical findings in the research of DD and ADHD implicate abnormalities in cortico-striatal pathways in both disorders [64, 99]. In DD, cortico-striatal disruption [10, 51, 76, 103] is presumed to affect the ability to acquire skills, procedures and stimulus–response associations acquired incrementally [24, 65, 97, 98]. Since language learning critically depends upon these domain general abilities [21, 97], impaired striatal-based learning is presumed to disrupt the typical course of reading, writing, and spelling skills in those with DD. In ADHD anatomical and functional abnormalities within the striatum [11] have been suggested to give rise to impulsive behaviors [45] and neurobiological models of ADHD posit that the deficit in striatal-based learning and memory is likely to arise from dopamine dysfunction within the neostriatum [78]. Recent evidence points to the right caudate as a shared neural substrate that is likely to be affected in both disorders [64].

## Reinforcement learning

The cortico-striatal network is responsible for reinforcement learning (RL), the process in which individuals learn by trial and error to make choices that exploit the likelihood of rewards and minimize the occurrence of penalties [91]. Therefore, based on the notion of cortico-striatal abnormalities in both disorders, RL is likely to be affected as well. Consistent with this assumption, RL deficits have been documented in DD [38, 42, 63, 72, 88] as well as in ADHD [35, 39, 49, 61, 95]. Impairments have been observed across RL tasks involving probabilistic feedback such as the Probabilistic Selection Task [35, 63] and the Weather Prediction Task [39, 42]. Furthermore, both DD and ADHD individuals are impaired in learning information integration categories [49, 88] which are believed to be acquired via striatal-based RL mechanisms [3]. Finally, both DD [38] and ADHD individuals [41] are impaired in probabilistic RL tasks when task conditions favor striatal-based memory engagement rather than hippocampal-based memory engagement, similar to a pattern observed among patients with striatal dysfunction [30, 33]. Notably some studies revealed intact RL in ADHD, but such findings are mostly found is tasks in which feedback is deterministic [47, 61] or in studies using relatively simple tasks with low number of stimuli [14, 48, 58].

## Model-free vs. model-based RL

Nevertheless, we still do not have a clear understanding of RL phenomena in both DD and ADHD or whether they are characterized by distinct/shared RL mechanisms. Recent advances in the field of neurocomputational models of cognition suggest that RL cannot be considered a unitary phenomenon. Rather, people employ different computational strategies when solving RL problems. One of these involves learning stimulus–response contingencies which, after formation, are less sensitive to outcome and reward (Yin & Knowlton, 2006). A more prevalent account of learning describes goal-oriented learning by focusing on learning outcome-action contingencies. Here, outcome-action contingencies can be based solely on recent history and presumed to arise computationally from model-free (MF) learning. The MF system learns the expected value of actions through prediction errors, which quantify the difference between the worth of actual and expected outcomes. In addition, action-outcome contingencies can be updated through model-based (MB) RL, which operates by learning a predictive model of multiple world states and action-outcome probabilities, and updating action-outcome contingencies by incorporating this information and planning an action course by using this model to evaluate the different outcomes prospectively over multiple future world states [13, 15, 20, 107]. Here MB is likely to involve learning state values based on planning processes [100].

It has been shown that animals and humans use a mixture of RL processes [13, 15, 20, 107, 111]. Limiting computational resources by concurrent task [66, 67] or inducing stress [66, 67] hinders MB but not MF learning, somewhat in line with observations that learning based on stimulus–response associations is resistant to distraction [32, 109]. The ability to use MB strategies follows a developmental trajectory, as in children MF learning is more dominant than MB learning [15]. Furthermore, MF learning has been shown to be sensitive to core components of executive functions, such as working memory and cognitive control [66–68]. Finally, in psychiatric disorders there is an imbalance between the ability to use MF vs. MB learning, such that those who have disorders associated with compulsivity and impulsivity tend to be impaired in their ability to use MB learning strategies [43, 102]. Neurobiologically, these two types of learning strategies are presumed to rely upon partially distinct neural substrates within the basal ganglia. It has been suggested that the dorsal lateral striatum subserves MF learning whereas the dorsal medial striatum underlies MB learning [44]. Despite this evidence, however, hippocampal damage in humans hampers MB learning but not MF learning [101]. Furthermore, although basal ganglia dopamine levels affect stimulus-response learning and hence are likely to affect MF learning [29], recent evidence points to the possibility that basal ganglia dopamine levels influence the ability to use MB but not MF learning strategies [82]. Notably, however, computational stimulations reveal that tonic dopamine levels influence the exploitation-exploration behavior trade-off when learning values is based on previous reinforcement history [50].

## The present study

The purpose of the present study was to examine RL behavior in two of the most common yet very different neurodevelopmental disorders. The theoretical and empirical body of research points to cortico-striatal abnormalities in both disorders (for a review see [99], which may lead to RL difficulties. RL has been studied in both ADHD and DD, but there has been no attempt to dissociate between different types of RL processes. Although a previous study revealed that methylphenidate increased risk taking in people with ADHD [62], we are aware of no studies that directly examined MB vs. MF RL learning in ADHD or DD. Likewise, there has been little attempt to compare RL in these neurodevelopmental disorders. The two-step task (TST; [13]) represents a recently popular approach to creating a task that differentiates between MF learning and MB processes and has been tested in a substantial number of studies in humans (e.g., [18, 66, 67, 82, 102, 106, 107]). In this task, a participant is required to make two decisions, each taking him closer to the outcome stage where a reward is revealed. TST allows a differentiation between two types of computations that may lead to impairments in reward-oriented behavior. The first is the MF effect of outcome on decisions, by which actions that were rewarded may not be sufficiently enhanced or associated with reward, leading to a weak association between actions and rewards. The second is the MB effect in which the likelihood that a path will lead to a reward is learned. Here, participants may not incorporate the probabilities of moving from one state to the next into their planning and decisions in the first step. Such computations, MF association and MB planning, may be uniquely disturbed in DD and ADHD.

Krishnan et al. [53] argued that cortico-striatal dysfunctions have been noted in both language and psychiatric disorders (such as ADHD) and raised the possibility that different computational models may explain the behavioral learning profile in each disorder. They specifically speculated that in developmental language disorders compared to psychiatric disorders (including ADHD) learning impairments will be less apparent when learning state values (the overall reward that one expects when choosing the state as the starting point). However as learning state values is common in MF and MB learning [90], learning state values based on planning processes may distinguish between language and attentional disorders. This notion is consistent with ample evidence showing that those with ADHD, but not those with DD, exhibit planning deficits and prefer immediate small rewards to delayed larger rewards [5, 16, 79, 85, 95]. Therefore, one could predict that MB learning will be selectively disrupted in ADHD. On the other hand, deficits in the MF association are likely to be impacted in both disorders, as shown by evidence pointing to an impaired ability to learn reinforcement contingencies in DD based on recent history [42, 63, 88] and ADHD [35, 39, 49, 61, 95].

## Results

### Data analysis

#### Power analysis

To determine whether the current study was adequately powered, we performed an a priori power analysis. Based on prior research, we computed an effect size of *d* = 0.65 for the key group difference in model-based learning [82]. Using the software package G*Power [23] with power (1 − β) set at 0.80 and α = 0.05, one-tailed, we determined that a sample size of 30 per group was required. Thus, the current study was adequately powered.

#### Screening

We excluded individuals who stayed with the same response-key for more than 95% of the trials (0 were excluded) or had more than 25% implausible quick reaction-times in either the first or second stage (< 150 ms; 1dys, 4 ADHD were omitted). For the remaining respondents we omitted from analysis trials with implausible reaction times (< 150 ms), and the first trial in the task (2.48%).

#### Modal based vs. model free learning

Each clinical population group (DD/ADHD) was tested against its own control group (neurotypcials matched to the DD group and neurotypicals matched to the ADHD group, respectively) and each clinical and control group were matched by age, gender, and non-verbal intelligence. Analyses were performed using *R* (Team, 2020). Mixed-effect logistic regression models were conducted using the lme4 package [8]. For both experiments we used the following analyses:

To assess whether the groups differed in their ability to use MF vs. MB strategies, we evaluated the effect of events on each trial (trial n) on the first-step decision in the subsequent trial (trial n + 1). The two key predictors in trial n were whether or not a reward was received and whether this occurred after a common or rare transition to the second stage. We evaluated the impact of these events on the chance of repeating the same first-stage choice in trial n + 1. A pure model free agent is likely to repeat a first-stage choice that results in reward regardless of the previous transition type, predicting a positive main effect of reward on first-stage stay probabilities. A pure model-based agent, on the other hand, evaluates first-stage actions in terms of second-stage alternatives they tend to lead to. To examine the contribution of these two systems (i.e., MF vs. MB) we calculated a mixed effect logistic regression, where previous outcome (rewarded vs. unrewarded), previous transition (rare vs. common), group (DD/ADHD vs. control), and all related interactions were entered as fixed effects predicting the probability that the participant would repeat the same choice (stay probability). We further included in this analysis (and in all further mixed-effects regression analyses), a random effect of participants on the intercept parameter [31].

As an additional measure of model-based abilities, we analyzed second-stage reaction times (RTs) as a function of transition (rare vs. common). A previous study showed that greater deployment of model-based strategies in the first stage led to shorter RTs after common vs. rare transitions [81]. Thus, the effect of transition on second-stage RTs can serve as an additional estimate for model-based involvement [12, 17]. We calculated a mixed effect linear regression, where transition (rare vs. common) and group (DD/ADHD vs. control) were entered as fixed effects predicting second-stage RTs. The regression included an additional random effect of participants on the intercept parameter.

## Experiment 1: DD vs. controls

### First stage MF vs. MB effects

**Fig. 1:**
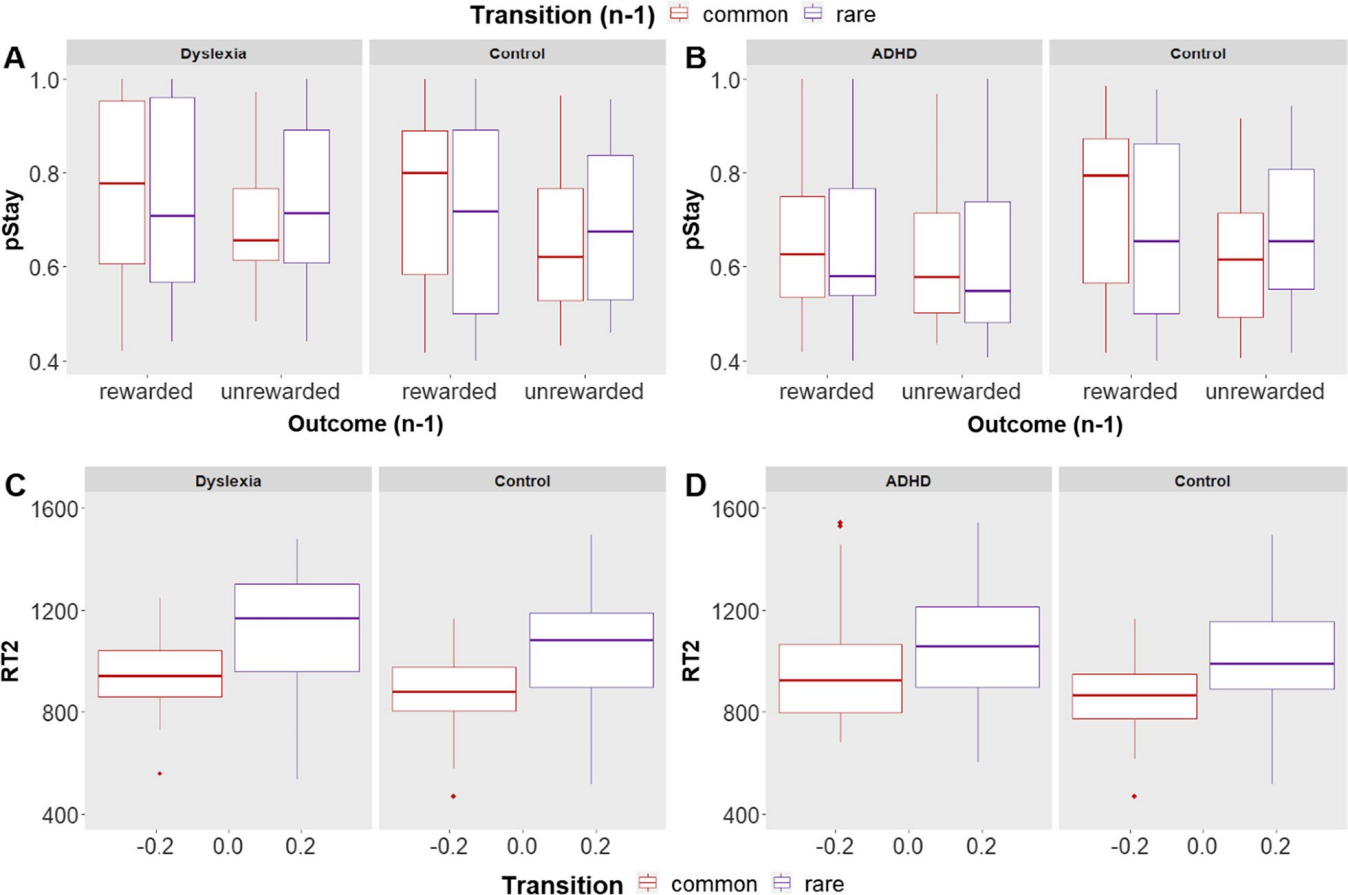
Performance of DD/ADHD and controls on the two-step task. **A**, **B** Y-axis represents the probability of repeating the same first-stage choice as a function of the transition in the previous trial (common versus rare) and of the outcome (rewarded versus unrewarded). **C**, **D** Y-axis represents second-stage reaction times (RTs) as a function of transition (rare vs. common) and group (DD/ADHD vs. controls)

Table 1 shows the results of this model and Fig. 1A illustrates the effects. We observed a significant main effect of previous outcome [*χ2* (1) = 114.61, *p* < 0.001] on participants’ choices, showing that participants were more likely to stay with their first-stage choice when the previous trial was rewarded vs. unrewarded, across groups. This effect is indicative of model-free learning across groups. We further found that group modulated this effect, as evident by a significant previous outcome × group interaction [χ2 (1) = 8.08, *p* = 0.004], such that the DD group showed a smaller influence of previous outcome on first-choice stay probability. We also observed a significant previous outcome × previous transition interaction, [*χ2* (1) = 13.424, *p* < 0.001], indicative of model-based learning. The three-way interaction of reward × transition × group was not significant [*χ2* (1) = 1.52, *p* = 0.21], suggesting that people with DD tended to evaluate first-stage actions in terms of the second-stage alternatives associated with them, similar to how neurotypicals evaluated them.

**Table 1:** Results of the mixed-effects model of first-stage MF and MB effects

	Chisq	Df	Pr (> Chisq)	CI (95%)	
Reward_oneback	114.62	1.00	< 2.2e-16	− 1.12	− 0.30
Transition_oneback	1.69	1.00	0.19	− 0.41	0.21
Group	1.63	1.00	0.20	− 1.04	0.49
Reward_oneback:transition_oneback	13.42	1.00	0.00	− 0.21	0.71
Reward_oneback:group	8.08	1.00	0.00	− 0.69	0.43
Transition_oneback:group	0.99	1.00	0.32	− 0.55	0.21
Reward_oneback:transition_oneback:group	1.52	1.00	0.22	− 0.42	0.77

### Second-stage MB effect

Table 2 shows the results of this model and Fig. 1C illustrates the effects. We found a significant main effect of transition [χ2 (1) = 611.35, *p* < 0.001], where choices following a rare transition were slower than those following common transitions. None of the remaining effects with group were significant. This observation is consistent with the finding that those with DD did not differ from matched neurotypicals in their ability to use MB strategies.

**Table 2:** Results of the mixed-effects model of RT

	Chisq	Df	Pr (> Chisq)	CI (95%)	
Transition	611.35	1.00	< 2e-16	126.10	232.26
Group	2.67	1.00	0.10	− 142.31	9.89
Transition:group	0.09	1.00	0.76	− 76.84	73.47

## Experiment 2: ADHD vs. controls

### First-stage MF vs. MB effects

Table 3 shows the results of this model and Fig. 1B illustrates the effects. We observed a significant main effect of previous outcome [χ2 (1) = 92.603, p < 0.001], indicative of model-free learning across groups. However, group modulated this effect, as evident by a significant previous outcome × group interaction [*χ2* (1) = 8.077, *p* = 0.01], such that the ADHD group showed a smaller influence of previous outcome on first-choice stay probability. We also observed a significant previous outcome × previous transition interaction [χ2 (1) = 15.967, *p* < 0.001], indicative of model-based learning. The triple interaction of reward*transition*group was significant [χ2 (1) = 4.755, *p* = 0.029], such that ADHD participants exhibited a reduced MB behavior (i.e., smaller previous outcome × previous transition interaction) compared to neurotypicals.

**Table 3:** Results of the mixed-effects model of first-stage MF and MB effects

	Chisq	Df	Pr (> Chisq)		CI (95%)
Reward_oneback	92.60	1.00	< 2.2e-16	− 0.39	− 0.12
Transition_oneback	4.17	1.00	0.04	− 0.29	0.05
Group	0.07	1.00	0.80	− 0.20	0.67
Reward_oneback:transition_oneback	15.97	1.00	0.00	− 0.15	0.35
Reward_oneback:group	8.08	1.00	0.00	− 0.53	− 0.15
Transition_oneback:group	0.10	1.00	0.75	− 0.47	0.02
Reward_oneback:transition_oneback:group	4.75	1.00	0.03	0.07	0.77

### Second-stage MB effect

Table 4 shows the results of this model and Fig. 1D illustrates the effects. We found a significant main effect of transition [χ2 (1) = 340.94, *p* < 0.001], where choices following a rare transition were slower than those following common transitions. Importantly, there was a significant transition by group interaction [χ2 (1) = 29.551, *p* < 0.001], such that the transition effect (slower responses in rare compared to common states) was higher in the control group compared with the ADHD group, consistent with lower deployment of model-based strategies in the first stage for the ADHD compared to the control group. To test whether both groups exhibited a transition effect despite the differences in magnitude of the effect as indicated by the interaction, pairwise contrasts were calculated using the emmeans function from the emmeans package [60]. Two pairwise contrasts for the levels of Transition (rare vs. common) were calculated for each group using the output of emmeans as input for the function contrast together with the Bonferroni correction for multiple comparisons. The effect of transition (slower responses in rare cases compared to common states) was significant for both groups (ADHD: estimate = 88.8, SE = 27.9, z. ratio = 3.18, p = 0.0015; TD: estimate = 173, SE = 27, z. ratio = 6.410 p < 0.001).

**Table 4:** Results of the mixed-effects model of RT

	Chisq	Df	Pr (> Chisq)		CI (95%)
Transition	340.95	1.00	< 2.2e-16	35.26	142.36
Group	2.67	1.00	0.10	− 203.55	− 6.63
Transition:group	29.55	1.00	0.00	6.45	157.96

## General discussion

RL impairments have been implicated in both DD and ADHD [35, 39, 42, 49, 61, 63, 88, 95]. Here, we aimed to determine how different RL types (MF vs. MB) are affected in these two most common yet different neurodevelopmental disorders, and whether shared and distinct learning profiles could be observed across the two disorders. Consistent with previous studies, neurotypical participants in both Study 1 and 2 exhibited a typical use mixture of MF and MB strategies in the two-step task. However, the performance of young adults with DD and ADHD differed relative to matched neurotypicals.

Our results show that compared to matched controls, individuals with DD and individuals with ADHD were less likely to repeat a choice that was rewarded compared to neurotypicals. However, those with ADHD but not those with DD were less affected by MB considerations in their decisions compared to neurotypicals. Supporting this observation, those with ADHD but not those with DD exhibited reduced expectation violation effects, as reflected by a reduced RT difference between common and rare transitions as another indication of lower MB learning.

The observation of impaired model-based RL in ADHD is consistent with previous findings showing that the ability to use MB strategies is disrupted in disorders characterized by striatal dopamine dysfunction, such as Parkinson’s disease [82] and broadens it to populations that are also associated with striatal dopamine alterations and impulsive tendencies, such as ADHD. The results are especially consistent with previous findings showing temporal discounting in those with ADHD [5, 16, 79, 85, 95]. The impaired ability of people with ADHD to use MB strategies could arise from several reasons: First, ADHD participants can have difficulties/are slower at generating complex internal models of task environments. Another possibility is that they are able to generate internal models but fail to exert the cognitive effort required to follow these mental models. Finally, it can be the case that MB learning is overwhelmed by the absence of automatic control routines that are normally provided by the MF system, rendering MB learning less effective in ADHD. The latter possibility, however, is inconsistent with the results of the DD group that demonstrated preserved MB learning despite impaired reward effect relative to neurotypicals. Future studies are undoubtedly needed in order to understand the reduced model-based behavior we observed in those with ADHD. The observation of impaired MF and MB learning in ADHD is consistent with neurobiological models of ADHD positing impaired RL mechanisms [35, 78, 96]. Although these models differ in their level of explanation [60] all assume that RL processes are likely to be impaired in ADHD. The present findings add to this theoretical body of research by pointing to the possibility that RL deficits in ADHD cannot be conceived as a unitary phenomenon but that two distinct types of RL processes are likely to be affected in this disorder. Despite differences in the ability to use MB strategies in the ADHD and DD groups, a similar previous-outcome main effect impairment was observed in both groups compared to neurotypicals. There are several possibilities for explaining the reduced previous-outcome main effect we observed in the two groups. First, such an effect could be explained by noise or an increased tendency to explore the environment [92], which could reasonably be associated with decreased use of MF strategies [28]. This possibility is consistent with recent findings showing that ADHD symptoms are negatively correlated with win-stay scores [74]. Indeed, computational stimulations reveal an effect of altered dopamine levels on the exploration-exploitation trade-off. As such, altered dopamine levels in ADHD could give rise to such trade-off, consistent with neurobiological models of ADHD [35, 78, 96]. Notably, increased exploration in DD is less consistent with recent findings showing similar win-stay and lose-shift scores in DD compared to neurotypicals in a probabilistic reinforcement learning task [63]. Another possibility is that the ability to learn reinforcement contingencies based on the recent outcome history is more disrupted in neurodevelopmental disorders compared to typical populations [35, 39, 42, 49, 61, 63, 88, 95]. In this regard, some have speculated that MF learning has notable parallels with procedural learning and that hippocampal-based learning is more equivalent with model-based behavior [19]. Considering this, the present results resonate with theoretical models positing a procedural learning dysfunction in DD alongside intact hippocampal-based learning abilities [65, 98, 99]. Furthermore, at first glance the observation of impaired MF and MB learning in ADHD is inconsistent with theoretical and empirical research positing impaired striatal-based learning in ADHD alongside spared hippocampal-based learning [6, 41, 45, 99]. However, MB learning is also likely to involve additional neural substrates and in particular the dorsolateral prefrontal cortex [86], which has been shown to be affected in ADHD [27]. Therefore, it can be the case that RL that rely on the dorsolateral prefrontal cortex as well are more likely to be affected in ADHD [49], rather than RL that are mostly associated with greater activation in hippocampal-based structures [41]. Further studies are required to explore this possibility.

A further major contribution of the present study to previous literature is the examination of types of strategies employed by participants with DD during learning. The results of the present study suggest that learning deficits observed in DD might arise from impaired efficiency in using MF-based strategies. Our study therefore highlights the importance of studying not only learning deficits in DD but also use of strategies that might have a role in them. Since rule-based learning may be analogous to MB RL and procedural-based strategy may be analogous to model-free RL [68], the ability to use procedural-based strategies should be selectively disrupted in DD consistent with recent observations (Gabay, Roark & Holt, [112]). Procedural learning plays an important role in language acquisition [97] including the ability to form sound categories [26, 55]. Impaired category learning via procedural learning mechanisms could therefore influence the ability of people with DD to form precise phonological representations with negative effects on reading and phonological skills [40].

Taken together, the present findings reveal an interesting dissociation between attentional and language developmental disorders. A common deficit in MF association may lead to learning impairments in both disorders. Such impairments may be related to attenuated effect or detection of outcome valance, or to problems in associating the reward with its preceding actions, especially linking it to actions that are twice removed from the outcome (first-stage decisions). However, the two disorders show different effects of MB mechanisms. While the DD group showed an intact MB representation of the path leading to outcome and the ability to dynamically use this information when making planning decisions, i.e., thinking ahead, ADHD participants did not incorporate this information. This may be because of inappropriate representation of transition probability (i.e., of the path) or by failing to incorporate this information in decisions. This distinction between planning ahead and updating backwards may be a characteristic of other deficiencies between these two disorders, to be explored in future studies, and may call for different interventions. Such findings could be interpreted in light of the multiple deficit model of developmental disorders, according to which every developmental disorder involves multiple cognitive risk factors [70]. Based on this notion, it may be the case that impairments in model-free RL may be one of the key risk factors for DD and ADHD [71] but that the MB learning deficit is related to the defining neuropsychological features of ADHD but not of DD.

The two-step task is one of the most common paradigms that has been suggested to differentiate between MF learning and MB processes and has been tested in a substantial number of typical and impaired populations. Nevertheless, caution is warranted in interpreting behavioral performance in this task, as several modifications to this paradigm could affect the relative contribution of each system to behavior. For example, it has been shown that MF RL can produce behavioral patterns in the two-step task that could be interpreted as MB RL [2]. Furthermore, providing explicit instructions led participants to make primarily model-based choices with little model-free influence [25]. However, in the current study, we found that ADHD and DD showed distinctive deviation from the behavior of control participants in the same task. This suggests that, to some extent, the two-step task used here can differentiate between learning processes and provide an informative insight into how such learning processes are impaired in different neurodevelopmental disorders. It will be important to direct future investigations to examining variants of the two-step task in ADHD/DD in order to more precisely understand the nature of MF/MB processes in these neurodevelopmental conditions.

To conclude, in the present study we compared different types of RL across DD and ADHD participants and their matched controls. Our results show a shared cognitive deficit in MF learning across participants with DD and ADHD relative to neurotypicals, alongside a deficit in MB learning that was selectively disrupted only in the ADHD group. These results suggest that distinct RL profiles can distinguish between language and attentional disorders.

## Methods

### Experiment 1: Participants with DD and neurotypical participants

Sixty-six university students (35 with DD, 15F and 31 controls, 18F) took part in the study. All participants were university students in Israel, from families with middle to high socioeconomic status. All participants were screened for being native Hebrew speakers, had no history of neurological disorders and/or psychiatric disorders, had normal or corrected-to-normal vision and normal hearing. The inclusion criteria for the DD group was (1) a formal diagnosis by a licensed clinician; (2) the absence of a formal diagnosis of attention deficit hyperactivity disorder (ADHD) or a specific language impairment; (3) a score below the clinical cutoff on the adult ADHD self-report scale (ASRS); (4) a score below a 1SD local norm cut-off for phonological decoding [108]; (5) a cognitive ability score within the normal range > 10th percentile Raven score [75]. Based on these criteria, three participants with DD were excluded from the final sample. The control group was composed of individuals with no history of learning disabilities who exhibited no difficulties in reading (e.g., were above the reading cutoff (non-word reading) and was matched in age, gender, and nonverbal intelligence (assessed by the Raven test) to the DD group. The Institutional Review Board of the University of Haifa approved the study (no. 18/099), which was conducted in accordance with the Declaration of Helsinki, with written informed consent provided by all participants. Participants received a compensation of NIS 120 (approximately $37) for participating in the study.

Participants underwent a series of cognitive tests (Table 5) to evaluate basic cognitive ability, assessed by the Raven test [75] as well as tests of verbal short-term memory (Digit span test; Wechsler, 1997 [104]), rapid automatized naming skills (RAN tests;[9], phonological processing (phoneme segmentation, phoneme deletion, and Spoonerism), reading skills [83, 84], and attentional functions (ASRS; [52].

**Table 5:** Psychometric Tests

Ability	Test	Description
INTELLECTUAL ABILITY	Raven (Raven, Court, & Raven, 1992)	This test is designed to assess nonverbal intelligence. Participants are required to choose an item from the bottom of the figure that will complete the pattern at the top of the figure. The maximum raw score for this test is 60. The test reliability coefficient is .9
VERBAL SHORT-TERM MEMORY	Digit Span Wechsler Adult Intelligence Scale (WAIS-III; [104])	In this task, participants are required to recall the numbers presented auditorily in the order they were presented by the examiner. The maximum total raw score is 28. Task administration is discontinued after a failure to recall two trials with a similar length of digits. The test reliability coefficient is .9
DECODING	One-minute test of words and One-minute test of nonwords [83]	These tests aim to assess reading skills. The one-minute test of words contains nonvowelized words of an equivalent level of complexity. The one-minute test of nonwords contains increasingly complex vowelized nonwords. Each test requires the participant to read aloud as quickly and accurately as possible within one minute. The maximum raw score for the one-minute test of words is 168. The maximum raw score for the one-minute test of nonwords is 86
PHONOLOGICAL PROCESSING	Phoneme Deletion [9]	In this test, participants are required to repeat nonwords without a specific phoneme as rapidly as possible. The nonwords are presented auditorily and vary in complexity, with a maximum total raw score of 25
	Phoneme segmentation test [9]	This measure assesses the participant's ability to break a word into its component phonemes. For example, the word *fo* has two phonemes */f/ /o/*. The maximum raw score is 16
	Spoonerism Task (developed by Peleg & Ben-Dror)	Participants are required to switch the first syllables of two word-pairs and then synthesize the segments to provide new words. The maximum raw score is 12
NAMING SKILLS	Rapid Automatized Naming (RAN) [9]	Participants are required to orally name items presented visually as rapidly as possible. The exemplars are drawn from a constant category (RAN colors, RAN categories, RAN numerals, and RAN letters). This requires retrieval of a familiar phonological code for each stimulus and coordination of phonological and visual (color) or orthographic (letter) information quickly on time. The reliability coefficient of these tests ranges from .98 to .99
ATTENTION	Adult ADHD Self-Report Scale (ASRS)	An 18-item questionnaire based on the DSM-IV criteria for identifying ADHD in adults. The questions refer to the past 6 months. The ASRS rating scale includes 0–5 rating (very often = 5 points, often = 4 points, sometimes = 3 points, rarely = 2 points, never = 1 point). A total score of more than 51 points is used to identify ADHD

These tests were used to assert group differences in reading and phonological abilities. The results, shown in Table 6, indicate that the groups did not differ in age, cognitive abilities, or attentional skills, but compared to the control group the DD group displayed a profile of reading disability compatible with the symptomatology of developmental dyslexia. This group differed significantly from the control group on both rate and accuracy measures of word reading and decoding skills. The DD group demonstrated deficits also in the three key phonological domains: phonological awareness (Spoonerism, phoneme segmentation, phoneme deletion), verbal short-term memory (digit span), and rapid naming (rapid automatized naming).

**Table 6:** Demographic and psychometric data of the DD and control groups

Measurement	Control	S.D	Dyslexia	S.D	t value	p
Age (in years)	25	2.828	25.29	3.579	− 0.354	0.724
Decoding						
Oral words recognition (accuracy)	118.838	15.132	71.967	22.443	9.641	.001
Oral words recognition (speed)	120.193	15.142	75.838	24.992	8.451	.001
Oral non-words recognition (accuracy)	63.903	11.344	25.258	9.774	14.369	.001
Oral non-words recognition (speed)	67.935	11.132	41.387	12.776	8.723	.001
Naming skills						
Naming letters (time)	21.774	2.883	25.258	3.759	− 4.094	.001
Naming objects (time)	32.548	4.945	41.032	7.259	− 5.378	.001
Naming numbers (time)	17.419	2.566	21.612	2.917	− 6.009	.001
Naming colors (time)	27.387	5.358	32.935	5.703	− 3.948	.001
Phonological processing						
Phoneme segmentation (time)	72.774	16.206	147.58	66.229	− 6.109	.001
Phoneme segmentation (accuracy)	15.032	0.982	11.935	3.829	4.362	.001
Phoneme deletion (time)	87.29	13.473	183.806	48.387	− 10.699	.001
Phoneme deletion (accuracy)	23.612	1.819	19.322	5.344	4.231	.001
Spoonerism (time)	109.064	22.196	270.193	113.185	− 7.778	.001
Spoonerism (accuracy)	18.741	1.389	15.29	4.54	4.047	.001
Short verbal working memory						
Digit span^a^	12.677	2.599	9.838	2.222	4.621	.001
Intellectual ability						
Raven test^a^	70.161	17.817	64.29	24.985	1.065	0.292
Attentional functions						
ASRS^a^	32.483	6.762	31.903	9.148	0.284	0.777

### Experimenet 2: Participants with ADHD and neurotypical participants

Sixty-five university students (35 with ADHD; 23F and 30 controls; 22F) took part in the study. All participants were university students in Israel, from families with middle to high socioeconomic status. All participants were screened for being native Hebrew speakers, had no history of neurological disorders and/or psychiatric disorders, had normal or corrected-to-normal vision and normal hearing. The inclusion criteria for the ADHD group included (1) a formal diagnosis of ADHD by an authorized clinician; (2) positive screening for ADHD based on the adult ADHD self-report scale (ASRS; [52], namely a score >  = 51; (3) the lack of a formal diagnosis of a comorbid developmental disorder such as developmental dyslexia; (4) a cognitive ability score within the normal range > 10th percentile Raven score. The control group was composed of individuals with no history of learning disabilities who exhibited no difficulties in attentional skills (e.g., did not receive a positive score of ADHD based on the ASRS) and was matched in age, gender, and nonverbal intelligence (assessed by the Raven test) to the DD group. The Institutional Review Board of the University of Haifa approved the study (no. 18/099), which was conducted in accordance with the Declaration of Helsinki, with written informed consent provided by all participants. Participants received a compensation of NIS 120 (approximately $37) for participating in the study.

All participants underwent a series of cognitive tests to evaluate general intelligence as measured by Raven’s SPM tests [75], as well as tests of attentional (ASRS; [52] and reading skills [83]. Details of the tests are presented in Table 5, and the results are shown in Table 7. The groups did not differ significantly in age, intelligence, or reading skills. Naturally, the ADHD group differed significantly from the control group in the ADHD measures derived from the ASRS questionnaire.

**Table 7:** Demographic and psychometric data of the ADHD and control groups

Measurement	Control	Std. Deviation	ADHD	Std. Deviation	t value	p
Age (in years)	25.2	3.01	24.33	3.844	0.972	0.335
Decoding						
Oral words recognition (accuracy)	112.966	14.919	107.466	13.415	1.501	0.139
Oral words recognition (speed)	114.966	14.48	109.766	13.317	1.448	0.153
Short verbal working memory						
Digit span^a^	11.633	2.326	9.833	2.52	2.875	0.006
Intellectual ability						
Raven test^a^	61.033	18.601	54	29.058	1.117	0.27
Attentional functions						
ASRS^a^	32.666	6.686	68.766	7.85	− 19.174	.001

### Two-step task

**Fig. 2:**
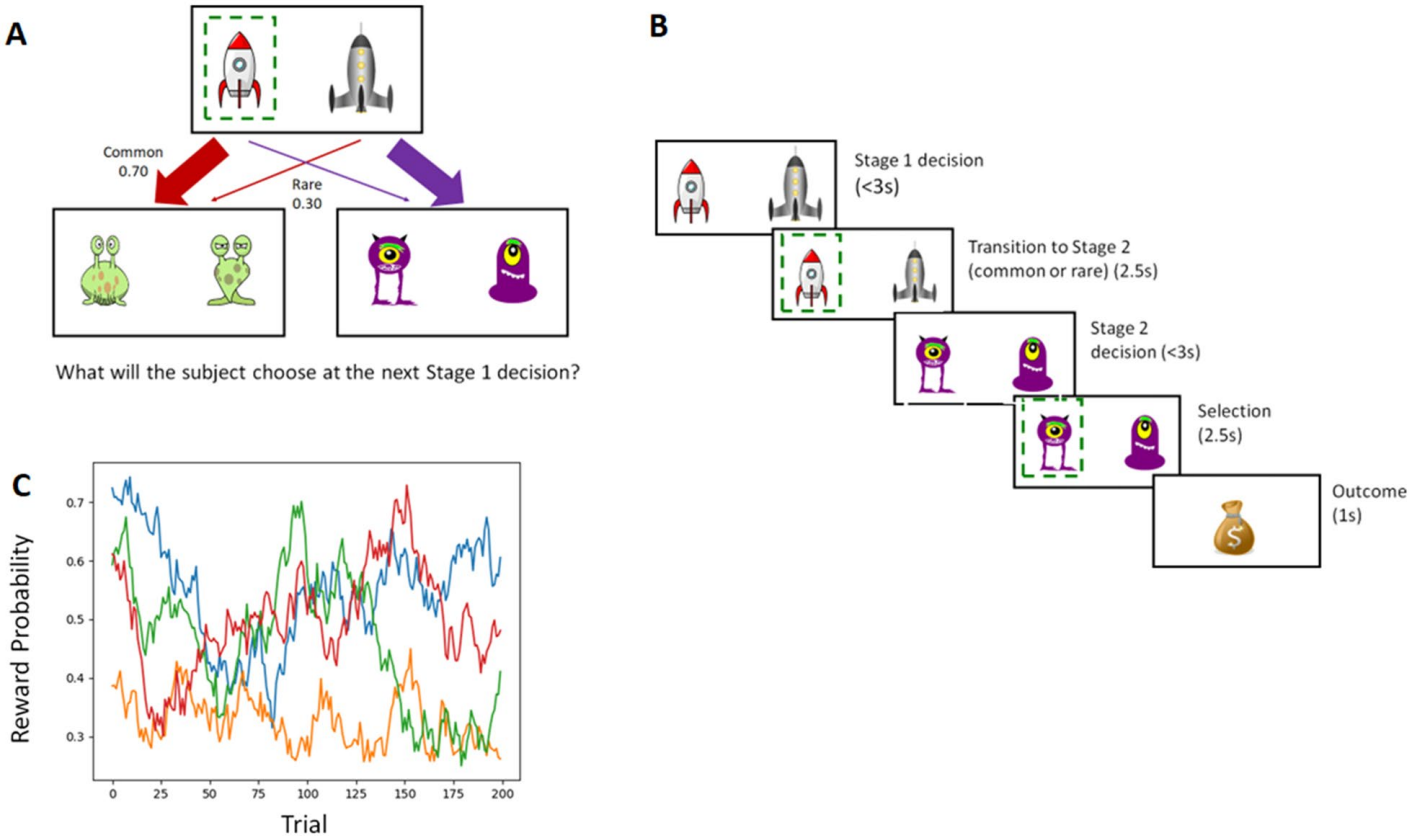
Two-step task designed to assess model free and model based learning. **A** Stage 1 was a choice between two spaceships. This choice determined the transition to the next stage according to a fixed probability scheme: each spaceship was predominantly associated with one or the other Stage 2 states (i.e. planets) and led there 70% of the time. In Stage 2. Participants selected one of two aliens to learn if they would be rewarded. Each alien was associated with a probability rewarded. Each alien was associated with a probability reward (range: 0.25–0.75) that changed gradually over time, based on Gaussian random walk. **B** Timing of stages within a single trial **C** Example of one of the sets of Gaussian random walks that determined the probability of reward at each of the four stage 2 options

The task was similar to that employed in the study conducted by [82]. Each trial was divided into two stages, each of which required a decision (see Fig. 2. In the first stage, a choice was made between two spaceships. Participants were told that these spaceships could fly to one of two different planets. Each spaceship would land more often on a specific planet (i.e., common transition; 70% chance, yet could also land on the alternative planet in a minority of trials (i.e., rare transition; 30% chance. In the second stage, participants were asked to decide between two aliens. The selection of each alien led probabilistically to a reward determined by independently drifting Gaussian random walks [standard deviation (SD = 0.025] with a lower boundary of 0.25 probability of reward and an upper boundary of 0.75, such that the probability of reward from any particular second stage option changed very slowly from trial to trial. Because the transition from the first stage choice to the second stage planet was stochastic, first stage choices allowed dissociating two learning strategies, either MF or MB.

### Procedure

The experiment consisted of two sessions. Participants completed a background questionnaire at home and were invited to complete the cognitive battery tests. In the second session, participants completed the two-step task. Sessions were conducted in a sound-attenuated booth in front of a 14-in laptop monitor.

## Data Availability

The datasets used and/or analyzed during the current study are available from the corresponding author on reasonable request.
